# Prognostic Preoperative Factors in Surgical Patients with Colorectal Liver Metastases: A Nationwide Multicenter Study

**DOI:** 10.1007/s12029-026-01424-y

**Published:** 2026-02-12

**Authors:** Lucas Alexander Knøfler, Jeanett Klubien, Peter Nørgaard Larsen, Jens Georg Hillingsø, Jakob Kirkegaard, Torsten Pless, Mogens Tornby Stender, Mette Lise Lousdal, Susanne Dam Nielsen, Hans-Christian Pommergaard

**Affiliations:** 1 Department of Digestive Diseases, Transplantation and General Surgery, Copenhagen University Hospital, Rigshospitalet, Copenhagen, Denmark; 2 Hepatic Malignancy Surgical Research Unit (HEPSURU), Department of Digestive Diseases, Transplantation and General Surgery, Rigshospitalet, Inge Lehmanns vej 7, Copenhagen, 2100 Denmark; 3 Viro-immunology Research Unit, Department of Infectious Diseases, Copenhagen University Hospital, Rigshospitalet, Copenhagen, Denmark; 4 Institute for Clinical Medicine, University of Copenhagen, Panum Institute, Copenhagen, Denmark; 5 Department of Surgery, Aarhus University Hospital, Aarhus, Denmark; 6 Department of Surgery, Odense University Hospital, Odense, Denmark; 7 Department of Surgery, Aalborg University Hospital, Aalborg, Denmark; 8 Department of Clinical Epidemiology, Aarhus University Hospital, Aarhus, Denmark; 9 Department of Infectious Diseases, Rigshospitalet, Copenhagen, Denmark; 10 Department of Surgery and Transplantation, Rigshospitalet Inge Lehmanns Vej 7, Copenhagen Ø, 2100 Denmark

**Keywords:** Survival analysis, Risk factors, Tumor burden, Registries, Treatment outcome

## Abstract

**Purpose:**

Colorectal cancer is a leading cause of cancer-associated death. Metastatic disease in the liver is common and is associated with severely diminished survival rates. Despite various treatment modalities, comprehensive long-term survival data following surgical interventions for colorectal liver metastases (CRLM) is limited.

**Methods:**

This nationwide multicenter study used data from the Danish Liver Cancer Group registry. Patients undergoing initial surgery, including hepatic resection, ablation, or both, were included. Survival analysis was conducted using the Kaplan-Meier estimator and Cox proportional hazards models to evaluate the prognostic impact of clinical preoperative factors. Multiple imputation was performed for missing values.

**Results:**

Among 2316 patients, median survival was four years, with 1-, 3-, 5-, and 7-year survival rates of 91%, 61%, 42%, and 32%, respectively. Advancing age per year, higher performance status, metastases exceeding 3 cm, and an increasing number of metastases significantly increased mortality risks. Multivariable analysis further refined the hazard ratios (HR) for age per year (HR 1.02), WHO performance status ≥ 2 (HR 1.50), diameter of largest metastasis in cm (HR 1.19) and number of metastases ≥ 3 (HR 1.51).

**Conclusion:**

This study highlights the importance of considering age, performance status, and tumor burden in the preoperative evaluation to improve clinical outcomes.

## Introduction

Colorectal cancer is the third most common cancer and the second leading cause of cancer-related deaths globally [1]. Metastatic disease in the liver is common, and current literature suggests that up to 29% will develop liver metastases within three years after the diagnosis of colorectal cancer [2]. When left untreated, the natural history of colorectal liver metastases (CRLM) in patients is bleak with a median survival of 7.5 months and a 5-year survival rate near zero [3, 4].

The treatment strategy is tailored within a multidisciplinary approach. In Denmark, nearly all patients with CRLM are referred to a multidisciplinary team (MDT) conference. Surgical options include hepatic resection, either single- or two-stage hepatectomy, as well as ablation therapy or a combination of the two [5]. Liver transplantation may be performed in highly selected patients [6].

In the literature, data on survival outcomes after surgical treatment of CRLM are mainly based on single-center studies [7]. Thus, an overview of the survival outcome of surgical treatment of CRLM on a large scale is lacking. In this nationwide study, we aimed to report the long-term survival and the prognostic impact of common preoperative factors known at the time of referral for the first surgical treatment of CRLM.

## Materials and Methods

### Study Design and Settings

Data for this nationwide study originated from the Danish Liver Cancer Group (DLGCD) maintained by The Danish Clinical Quality Program – National Clinical Registries (RKKP). Data from referrals were reported to DLGCD by the four university hospitals in Denmark where patients with CRLM are surgically treated. The database was established in 2012 and started collecting data in 2013.

DLGCD contains information from MDT referrals including patient and tumor characteristics, details on surgical treatment as well as follow-up data regarding date of death and date of censoring. Patients were followed from the date of the first surgical intervention, and until the date of death, censoring, or end of follow-up, 15th November 2021, whichever came first.

The study is reported in accordance with the Strengthening the Reporting of Observational Studies in Epidemiology (STROBE) guidelines [8]. Approval to conduct the study was granted by the Danish Data Protection Agency (P-2022-628). In accordance with Danish law, informed consent was not required for this study.

### Study Population

Patients registered in DLGCD and treated with hepatic resection, ablation, or a combination of the two as the first surgical intervention for CRLM were included. The database was manually screened, and duplicates in civil registration numbers were removed. Furthermore, patients residing outside of Denmark or with missing or ambiguous follow-up data were excluded. The database did not allow for a distinction between synchronous and metachronous liver metastases. Further, no information was available regarding the indication for the given treatment, treatment with chemotherapy, or characteristics and treatment of the primary tumor.

### Definitions of Covariates

Surgical treatment was categorized as hepatic resection, ablation, or a combination of the two. The diameter of the largest metastasis treated was categorized as ≤ 3.0 cm or > 3.0 cm. This was based on previous literature suggesting that metastases above this size may be insufficiently treated by ablation [9, 10]. Number of metastases was categorized as 1, 2, and ≥ 3. WHO performance status (PS) was categorized as PS of 0, 1, and ≥ 2.

### Statistical Methods

Statistical analyses were conducted with R 4.3.0 and RStudio. Statistical significance was defined as a p-value < 0.05. Descriptive statistics are reported as frequencies and percentages for categorical covariates, and medians with interquartile ranges (IQR) for continuous covariates.

Missing data for covariates regarding PS (*n* = 959 [41%]), size of largest metastasis treated (*n* = 916 [40%]), and number of metastases (*n* = 714 [31%]) were handled with multiple imputation by chained equations using the MICE package. The imputation model was based on 50 imputations using the predictive mean matching method. Available information regarding patient and tumor characteristics, as well as place of treatment and type of treatment, was used as predictors for the imputation model. Patients with missing data were assumed to be accurately represented by the cohort of patients with complete data.

The Kaplan-Meier estimator was performed to estimate the overall cumulative survival rate as a function of time after procedure. Survival rates at 1, 3, 5, and 7 years were reported along with the median survival rate.

Univariable pooled analyses based on the Cox proportional hazards regression were performed on the following covariates to identify independent mortality risk factors: age increase per year, PS, diameter of largest metastasis (cm), and number of metastases. Results are reported as hazard ratios (HR) and 95% confidence intervals (CI). Further, a multivariable pooled analysis was performed for all significant risk factors to model the effect of potential confounding factors on the instantaneous mortality risk.

The proportionality assumption governing Cox proportional hazards and linearity of the covariates included were tested with Martingale cumulative residuals using the R package Mets. The test was performed on the first 10 imputed datasets, to mitigate test variability between imputations. No violations of the proportionality assumption were identified for any of the covariates included in the multivariable analysis. Similarly, no significant linearity issue was detected for the covariate regarding patient age. Further, the discriminatory ability of the multivariable model was assessed and reported for the first 10 imputed dataset with Harrell’s concordance index.

## Results

### Patient and Tumor Characteristics

The baseline characteristics of the 2316 patients included in the study are detailed in Table 1. The median age of the cohort was 68 years (IQR 61–74). The majority underwent hepatic resection, most patients were male (63%), and 34% presented with PS 0 before surgery. The median number of liver metastases was 2 (IQR 1–4), and the median diameter of the largest tumor treated was 2.2 cm (IQR 1.5–3.3).

**Table 1:** Characteristics of 2316 patients with colorectal liver metastases. IQR = Interquartile range

Variables			Total *N*, (%)
Age (years)			
Median (IQR)			68 (61–74)
Sex			
Male			1465 (63)
Female			851 (37)
Hospital			
Aalborg University Hospital			226 (10)
Aarhus University Hospital			506 (22)
Odense University Hospital			561 (24)
Rigshospitalet			1023 (44)
Procedure			
Hepatic resection			1276 (55)
Ablation			607 (26)
Hepatic resection with ablation			433 (19)
Diameter of largest metastasis (cm)			
Median (IQR)			2.2 (1.5–3.3)
≤ 3 cm			1022 (44)
> 3 cm			378 (16)
Missing			916 (40)
Number of metastases			
Median (IQR)			2 (1–4)
1			631 (27)
2			342 (15)
≥ 3			629 (27)
Missing			714 (31)
WHO performance status			
0			776 (34)
1			516 (22)
≥ 2			65 (3)
Missing			959 (41)

### Overall Survival

**Fig. 1:**
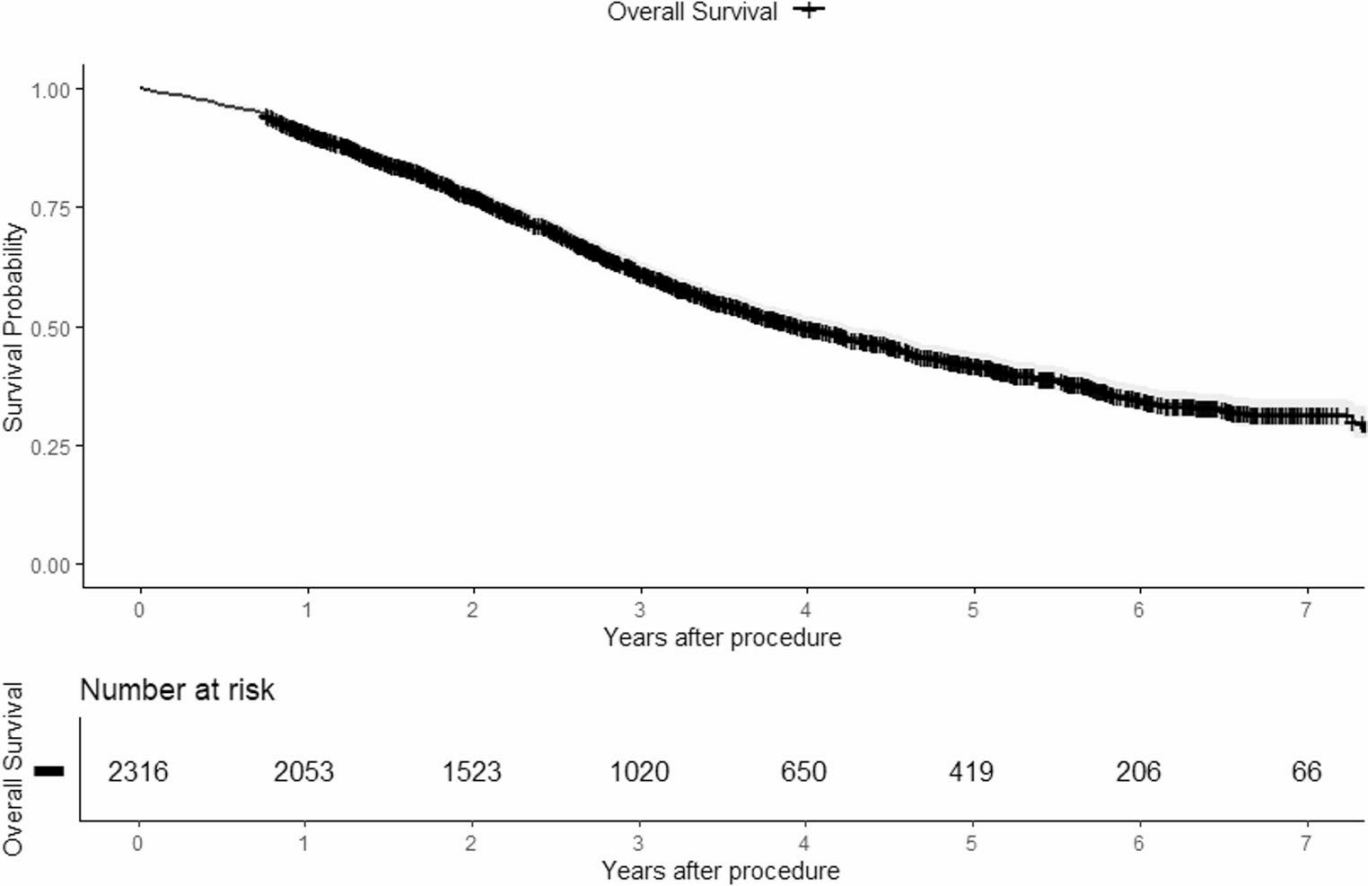
Kaplan-Meier estimator illustrating overall survival for 2316 patients treated with surgery for colorectal liver metastases

The median overall survival time was 4.0 years (95% CI 3.7–4.2), as shown in Fig. 1. The overall survival rates at one, three, five, and seven years were 91% (95% CI 90–92), 61% (95% CI 59–64), 42% (95% CI 40–45), and 32% (95% CI 29–35), respectively.

### Preoperative Prognostic Factors

Summarizes the pooled results from univariable and multivariable Cox proportional hazards regressions, analyzing mortality-associated preoperative factors after multiple imputation. Univariable analyses demonstrated significant associations between several factors and increased mortality risk. Specifically, each additional year of age was associated with a 1.02-fold increase in mortality risk (95% CI 1.01–1.02). A PS 2 or higher was associated with a 1.61-fold increase in risk (95% CI 1.26–2.05). Metastases exceeding 3 cm in diameter were linked to a 1.21-fold increase in mortality risk (95% CI 1.04–1.40), and the presence of three or more metastases was associated with a 1.45-fold increase in mortality risk (95% CI 1.18–1.77). Multivariable analysis confirmed that an increase in age by one year was significantly associated with a 1.02-fold increase in mortality risk (95% CI 1.01–1.02). Furthermore, tumor characteristics were significantly linked to a heightened mortality risk for metastases larger than 3 cm (1.19-fold increase, 95% CI 1.02–1.40) and for patients with three or more metastases (1.51-fold increase, 95% CI 1.27–1.79). A PS 2 or higher continued to show a significant association with a 1.50-fold increase in mortality risk. The association between PS 1 and risk of mortality as well as having 2 metastases did not reach statistical significance in the multivariable analysis. The concordance index for the multivariable model stood at 0.59 with a standard error of 0.009

**Table 2:** Univariable and fully adjusted multivariable Cox proportional hazards regression pooled analyses of common preoperative covariates in a cohort of 2316 surgically treated patients with CRLM. *Ref* reference group, *HR *hazard ratio, *CI* confidence interval

Variable		Univariable		Multivariable	
		HR (95% CI)	*P* value	HR (95% CI)	*P* value
Age					
Continuous		1.02 (1.01–1.02)	< 0.001	1.02 (1.01–1.02)	< 0.001
WHO performance status					
0		Ref.	-	Ref.	-
1		1.16 (0.98–1.37)	0.081	1.08 (0.89–1.31)	0.424
≥ 2		1.61 (1.26–2.05)	< 0.001	1.50 (1.12–1.89)	0.005
Diameter of largest metastasis					
≤ 3 cm		Ref.	-	Ref.	-
> 3 cm		1.21 (1.04–1.40)	0.013	1.19 (1.02–1.40)	0.030
Number of metastases					
1		Ref.	-	Ref.	-
2		1.24 (0.96–1.59)	0.095	1.22 (0.96–1.55)	0.110
≥ 3		1.45 (1.18–1.77)	< 0.001	1.51 (1.27–1.79)	< 0.001

## Discussion

This nationwide study assessed the role of several preoperative factors on risk of mortality and long-term survival following surgery for CRLM. Our analyses highlight that despite surgical interventions, the prognosis remains challenging. We demonstrated a significant association with increased mortality risk for age at surgery, performance status as well as diameter of the largest metastasis treated and the total number of metastases.

We found that patients undergoing surgery (resection, ablation, or a combination) had overall one-, three-, five-, and seven-year survival rates of 91%, 61%, 42%, and 32%, respectively, and a median overall survival time of 4.0 years. These rates align with those reported in another study on survival metrics for colorectal liver metastases [11]. Furthermore, our inclusion of combined resection and ablation procedures reflects modern parenchymal-sparing strategies. In a recent propensity-score matched analysis it was demonstrated that combined resection and thermal ablation yield comparable five-year survival to resection alone, while reducing the risk of postoperative liver failure [12].

Our findings suggest that age, in and of itself, does not impact mortality risk by more than 2% per year increase. However, adjusting for age profoundly affects the multivariable model by allowing comparison across age groups when assessing mortality risk for other preoperative factors. These findings suggest that while age alone should not be a contraindication for surgery, it should be considered alongside patients’ performance status and tumor burden. Previous studies align with this notion and highlights the need for careful patient selection and consideration of other risk factors [13–16]. Our findings are further supported by a recent Dutch nationwide study [17]. They demonstrated that while overall survival naturally decreases with age, the relative survival of some elderly patients was superior to younger cohorts, reinforcing the notion that chronological age alone should not be a contraindication for surgery. Several important considerations regarding age must be acknowledged. Chronological age is inherently imprecise, as it fails to account for variations in lifestyle, genetics, and environmental exposures. Recent studies in aging have highlighted the use of biomarkers, such as DNA methylation patterns, as a more accurate reflection of biological age [18]. Moreover, the progression of age is intrinsically linked to an increase in mortality risk. Despite these limitations, age remains a critical covariate in multivariable models, providing an estimate of the change in instantaneous mortality risk after surgery while adjusting for potential confounders, such as WHO PS.

The impact of PS on mortality risk and long-term survival, though commonly assessed in MDT operability evaluations, has been understudied. Our study supports the presence of PS in these assessments, showing a significant association with mortality risk even after adjusting for confounding factors.

Previous studies indicate that metastasis size is a crucial prognostic factor in both neoadjuvant and surgical contexts. Metastases larger than 3.0 cm may represent more aggressive disease and poorer prognosis [10, 16, 19, 20]. This study identified preoperative sizes of the largest metastasis >3 cm as a significant independent risk factor, and upon adjusting for confounding factors, patients exhibited a 19% increased mortality risk compared to those with metastases ≤ 3 cm.

Several studies have documented the adverse effects of having multiple metastases [16, 21]. Our data reinforce this notion, showing 22% increase in mortality risk for patients with 2 metastases and a 51% increase for patients with ≥ 3, although statistical significance was only achieved in the latter. We acknowledge that the size and number of metastases are parts of a larger puzzle, with potential effects of other tumor characteristics such as localization of the metastasis in the liver, resectability, synchrony with the primary tumor, and characteristics of the primary tumor which were unavailable for this study.

To our knowledge, this study is among the largest to date on this topic, utilizing, a population-based, nationwide clinical database with long and complete follow-up [16]. This approach reduces selection bias and provides a realistic sample from the clinical reality as compared to trials with strict inclusion criteria or other retrospective studies with very selected cohorts. The large size of the cohort further strengthens the survival estimates and prognostic value of the multivariable model. However, the study is limited by a high degree of missing data, addressed through multiple imputation models to approximate the missing values based on the characteristics of complete patient cases. Another important limitation is the lack of information on the use of chemotherapy and primary tumor characteristics. Chemotherapy has been shown to downstage initially unresectable tumors and improve long-term progression-free survival [22, 23]. Additionally, right-sided primary tumors are associated with worse survival following surgery for CRLM, likely due to their more aggressive tumor biology [24]. The absence of these factors constrains our ability to fully adjust the model estimates. Instead, our results should be viewed as reflecting a broad range of patient and disease characteristics.

## Conclusions

In conclusion, this study provides survival data reflective of modern surgical treatments for patients with CRLM. We found that age increase per year, PS ≥ 2, presence of three or more metastases as well as the diameter of largest metastasis treated > 3 cm were significant mortality risk factors. These identified factors may be valuable preoperatively in the general operability assessment of patients with CRLM.

## Data Availability

The registry data that support the findings of this study are available from the corresponding author upon reasonable request, subject to the successful attainment of the necessary legal and ethical approvals.
